# 3D-interologs: an evolution database of physical protein- protein interactions across multiple genomes

**DOI:** 10.1186/1471-2164-11-S3-S7

**Published:** 2010-12-01

**Authors:** Yu-Shu Lo, Yung-Chiang Chen, Jinn-Moon Yang

**Affiliations:** 1 Department of Biological Science and Technology, National Chiao Tung University, Hsinchu, Taiwan; 2 Institute of Bioinformatics, National Chiao Tung University, Hsinchu, Taiwan; 3 Core Facility for Structural Bioinformatics, National Chiao Tung University, Hsinchu, Taiwan

## Abstract

**Background:**

Comprehensive exploration of protein-protein interactions is a challenging route to understand biological processes. For efficiently enlarging protein interactions annotated with residue-based binding models, we proposed a new concept "3D-domain interolog mapping" with a scoring system to explore all possible protein pairs between the two homolog families, derived from a known 3D-structure dimmer (template), across multiple species. Each family consists of homologous proteins which have interacting domains of the template for studying domain interface evolution of two interacting homolog families.

**Results:**

The 3D-interologs database records the evolution of protein-protein interactions database across multiple species. Based on "3D-domain interolog mapping" and a new scoring function, we infer 173,294 protein-protein interactions by using 1,895 three-dimensional (3D) structure heterodimers to search the UniProt database (4,826,134 protein sequences). The 3D- interologs database comprises 15,124 species and 283,980 protein-protein interactions, including 173,294 interactions (61%) and 110,686 interactions (39%) summarized from the IntAct database. For a protein-protein interaction, the 3D-interologs database shows functional annotations (e.g. Gene Ontology), interacting domains and binding models (e.g. hydrogen-bond interactions and conserved residues). Additionally, this database provides couple-conserved residues and the interacting evolution by exploring the interologs across multiple species. Experimental results reveal that the proposed scoring function obtains good agreement for the binding affinity of 275 mutated residues from the ASEdb. The precision and recall of our method are 0.52 and 0.34, respectively, by using 563 non-redundant heterodimers to search on the Integr8 database (549 complete genomes).

**Conclusions:**

Experimental results demonstrate that the proposed method can infer reliable physical protein-protein interactions and be useful for studying the protein-protein interaction evolution across multiple species. In addition, the top-ranked strategy and template interface score are able to significantly improve the accuracies of identifying protein-protein interactions in a complete genome. The 3D-interologs database is available at http://3D- interologs.life.nctu.edu.tw.

## Background

A major challenge of post genomic biology is to understand the networks of interacting genes, proteins and small molecules that produce biological functions. The large number of protein interactions [1-3], generated by large-scale experimental methods [4-6], computational methods [7-13], and integrated approaches [14,15], provides opportunities and challenges in annotating protein functions, protein-protein interactions (PPI) and domain-domain interactions (DDI), and in modeling the cellular signaling and regulatory networks. An approach based on evolutionary cross-species comparisons, such as PathBLAST [16,17] and interologs (i.e. interactions are conserved across species [9,18]), is a valuable framework for addressing these issues. However, these methods often cannot respond how a protein interacts with another one across multiple species.

**Figure 1:**
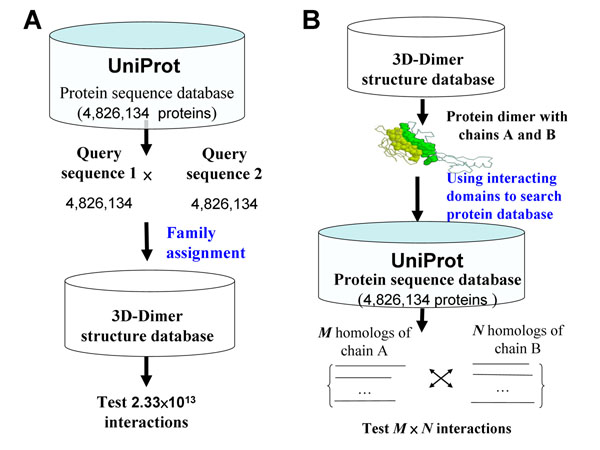
Two frameworks of template-based methods for protein-protein interactions (PPI). (A) For each query protein sequence pair, the method searches 3D-dimer template library to identify homologous templates for exploring the query protein pair, such as MULTIPROSPECTOR [20]. (B) For each structure in 3D-dimer template library, the method searches protein sequence database to identify homologous PPIs of the query structure, such as 3D-interologs.

Protein Data Bank (PDB) [19] stores three-dimensional (3D) structure complexes, from which physical interacting domains can be identified to study DDIs and PPIs using comparative modeling [11,20]. Some DDI databases, such as 3did [21], iPfam [22], and DAPID [23], have recently been derived from PDB. Additionally, some methods have utilized template-based methods (i.e. comparative modeling [11] and fold recognition [20]), which search a 3D-complex library to identify homologous templates of a pair of query protein sequences, in order to predict the protein-protein interactions by accessing interface preference, and score query pair protein sequences according to how they fit the known template structures. However, these methods [11,20] are time-consuming to search all possible protein-protein pairs in a large genome-scale database (Fig. 1A). For example, the possible protein-protein pairs on the UniProt database (4,826,134 sequences) are about 2.33×10^13^[24]. In addition, these methods are unable to form homologous PPIs to explore the protein-protein evolution for a specific structure template.

To address these issues, we proposed a new concept "3D-domain interolog mapping" (Fig. 1B): for a known 3D-structure complex (template T with chains A and B), domain *a* (in chain A) interacts with domain *b* (in chain B) in one species. Homolog families A' and B' of A and B are proteins, which are significant sequence similarity BLASTP *E*-values ≤10^-10^ and contain domains *a* and *b,* respectively. All possible protein pairs between these two homolog families are considered as protein-protein interaction candidates using the template T. Based on this concept, protein sequence databases can be searched to predict protein-protein interactions across multiple species efficiently. When the genome was deciphered completely for a species, we considered the rank of protein-protein interaction candidates in each species into our previous scoring system [13] to reduce a large number of false positives. The 3D-interologs database which can indicate interacting domains and contact residues in order to visualize molecular details of a protein-protein interaction. Additionally, this database can provide couple-conserved residues and evolutionary clues of a query sequence and its partners by examining the interologs across multiple species.

## Methods and materials

**Figure 2:**
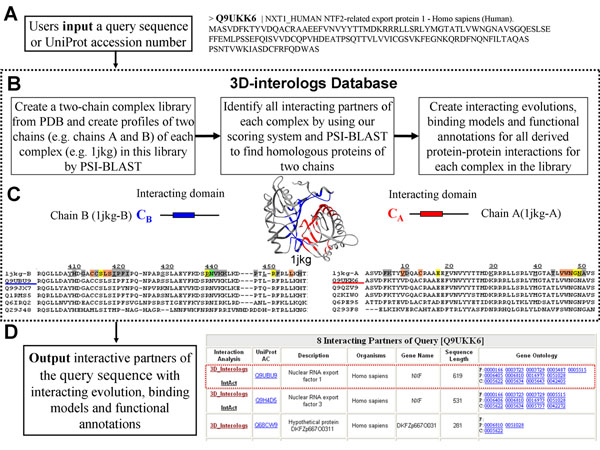
Overview of the 3D-interologs database for protein-protein interacting evolution, protein functions annotations and binding models across multiple species.

Figure 2 illustrates the overview of the 3D-interologs database. The 3D-interologs allows users to input the UniProt accession number (UniProt AC [24]) or the sequence with FASTA format of the query protein (Fig. 2A). When the input is a sequence, 3D-interologs uses BLAST to identify the hit interacting proteins. We identified protein-protein interactions in 3D-interologs database through structure complexes and a new scoring function using the following steps (Fig. 2B). First, a 3D-dimer template library comprising 1,895 heterodimers (3,790 sequences, called NR1895) was selected from the PDB released in Feb 24, 2006. Duplicate complexes, defined by sequence identity of above 98%, were removed from the library. Dimers containing chains shorter than 30 residues were also excluded [20,25]. Interacting domains and contact residues of two chains were identified for each complex in the 3D-dimer library. Contact residues, in which any heavy atoms should be within a threshold distance of 4.5 Å to any heavy atoms of another chain, were regarded as the core parts of the 3D-interacting domains in a complex. Each domain was required to have at least 5 contact residues and more than 25 interacting contacted-residue pairs to ensure that the interface between two domains was reasonably extensive. After the interacting domains were determined, its SCOP domains [26] were identified, and its template profiles were constructed by PSI-BLAST. PSI-BLAST was adopted to search the domain sequences against the UniRef90 database [24], in which the sequence identity < 90% of each other and the number of iteration was set to 3.

After 3D-dimer template library and template profiles were built, we inferred candidates of interacting proteins by 3D-domain interolog mapping. To identify the interacting-protein candidates against protein sequences in the UniProt version 11.3 (containing 4,826,134 protein sequences), the chain profile was used as the initial position-specific score matrix (PSSM) of PSI-BLAST in each template consisting of two chains (e.g. *C_A_* and *C_B_*, Fig. 2C). The number of iterations was set to 1. Therefore, this search procedure can be considered as a profile-to-sequence alignment. A pairing-protein sequence (e.g. S1 and S2) was considered as a protein-protein interaction candidate if the sequence identity exceeded 30% and the aligned contact residue ratio (CR) was greater than 0.5 for both alignments (i.e. S1 aligning to *C_A_* and S2 aligning to *C_B_*). For each interacting candidate, the scoring function was applied to calculate the interacting score and the *Z*-value, which indicates the statistic significance of the interacting score. An interacting candidate was regarded as a protein-protein interaction if its *Z*-value was above 3.0 and it ranked in the Top 25 in one species. The candidate rank was considered in one species to reduce the ill-effect of the out-paralogs that arose from a duplication event before the speciation [27]. These inferred interacting protein pairs were collected in the database.

Finally, for the hit interacting partner derived from 3D-domain interolog mapping, this database provides functional annotations (e.g. UniProt AC, organism, descriptions, and Gene Ontology (GO) annotations [28], Fig. 2D), and the visualization of the binding models and interaction evolutions (Fig. 2C) between the query protein and its partners. We then constructed two multiple sequence alignments of the query protein and its interacting partner (Fig. 2C) across multiple species. Here, the interacting-protein pair with the highest Z-score in a species was chosen as interologs for constructing multiple sequence alignments using a star alignment. The chains (e.g. Chains A and B, Fig. 2C) of the hit structure template were considered as the centers, and all selected interacting-protein pairs across species were aligned to respective chains of the template by PSI-BLAST. The 3D-interologs database annotates the important contact residues in the interface according to the following formats: hydrogen-bond residues (green); conserved residues (orange), conserved residues with hydrogen bonds (yellow) and other (gray).

### Data Sets

Two data sets were used to assess 3D-domain interolog mapping and the scoring functions. To determine the contribution of a residue to the binding affinity, the alanine- scanning mutagenesis is frequently used as an experimental probe. We selected 275 mutated (called BA-275) residues from the ASEdb [29] with 16 heterodimers whose 3D structures were known. Those mutated residues are contact residues and positioned at protein-protein interfaces. ASEdb gives the corresponding delta G value representing the change in free energy of binding upon mutation to alanine for each experimentally mutated residue. Residues that contribute a large amount of binding energy are often labeled as hot spots.

In addition, we selected a non-redundant set (NR-563), comprising 563 dimer protein structures from the set NR1895 to evaluate the performance of our scoring functions for predicting PPIs in *S. cerevisiae* and in 549 species collected in Integr8 database (2,102,196 proteins [30]).

### Scoring dunction and matrices

We have recently proposed a scoring function to determine the reliability of a protein- protein interaction [13]. This study enhances this scoring by dividing the template consensus score into the template similar score and the couple-conserved residue score. Based on this scoring function, the 3D-interologs database can provide the interacting evolution across multiple species and the statistic significance (*Z*-value), the binding models and functional annotations between the query protein and its interacting partners. The scoring function is defined as

**Figure 3:**
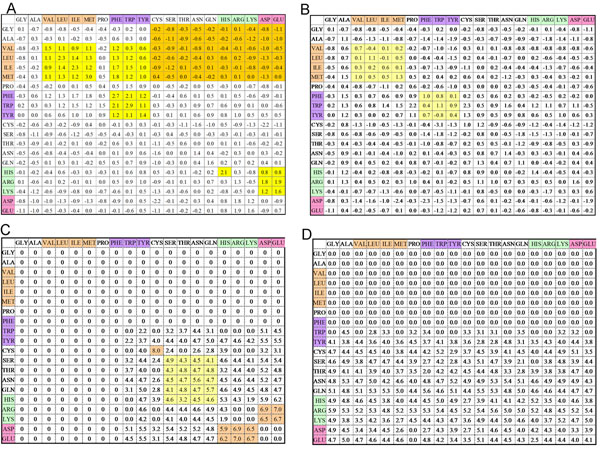
**Knowledge-based protein-protein interacting scoring matrices:** (A) sidechain-sidechain van-der Waals scoring matrix; (B) sidechain-backbone van-der Waals scoring matrix; (C) sidechain-sidechain special-bond scoring matrix; (D) sidechain- backbone special-bond matrix scoring. The sidechain-sidechain scoring matrices are symmetric and sidechain-backbone scoring matrices are nonsymmetric. For sidechain- sidechain van-der Waals scoring matrix, the scores are high (yellow blocks) if large-aliphatic residues (i.e. Val, Leu, Ile, and Met) interact to large-aliphatic residues or aromatic residues (i.e. Phe, Tyr, and Trp) interact to aromatic residue. In contrast, the scores are low (orange blocks) when nonpolar residues interact to polar residues. For sidechain-sidechain special- bond scoring matrix, the scores are high when an interacting resides (i.e. Cys to Cys) form a disulfide bond or basic residues (i.e. Arg, Lys, and His) interact to acidic residues (Asp and Glu). The scoring values are zero if nonpolar residues interact to other residues.

*E_tot_ = E_vdw_ + E_SF_ + E_sim_ + wE_cons_* (1)

where *E_vdw_* and *E_SF_* are the interacting van der Waals energy and the special interacting bond energy (i.e. hydrogen-bond energy, electrostatic energy and disulfide-bond energy), respectively; and *E_sim_* is the template interface similar score; and the *E_cons_* is couple-conserved residue score. The optimal *w* value was yielded by testing various values ranging from 0.1 to 5.0; *w* is set to 3 for the best performance and efficiency on predicting binding affinity (BA- 275) and predicting PPIs in *S. cerevisiae* and in 549 species (Integr8) using the data set NR- 563. The *E_vdw_* and *E_SF_* are given as

where *CP* denotes the number of the aligned-contact residues of proteins *A* and *B* aligned to a hit template; *Vssij* and *Vsbij* (*Vsbji*) are the sidechain-sidechain and sidechain-backbone van der Waals energies between residues *i* (in protein A) and *j* (in protein *B*), respectively. *Tssij* and *Tsbij* (*Tsbji*) are the sidechain-sidechain and sidechain-backbone special interacting energies between *i* and *j,* respectively, if the pair residues *i* and *j* form the special bonds (i.e. hydrogen bond, salt bridge, or disulfide bond) in the template structure. The van der Waals energies (*Vssij, Vsbij,* and *Vsbji*) and special interacting energies (*Tssij, Tsbij,* and *Tsbji*) were calculated from the four knowledge-based scoring matrices (Fig. 3), namely sidechain- sidechain (Fig. 3A) and sidechain-backbone van der Waals scoring matrices (Fig. 3B); and sidechain-sidechain (Fig. 3C) and sidechain-backbone special-bond scoring matrices (Fig. 3D).

These four knowledge-based matrices, which were derived using a general mathematical structure [31] from a nonredundant set of 621 3D-dimer complexes proposed by Glaser *et al.*[32], are the key components of the 3D-interologs database for predicting protein-protein interactions. This dataset is composed of 217 heterodimers and 404 homodimers and the sequence identity is less than 30% to each other. The entry (*S_ij_*), which is the interacting score for a contact residue *i, j* pair (1≤*i*, *j*≤20), of a scoring matrix is defined as  where *q_ij_* and *e_ij_* are the observed probability and the expected probability, respectively, of the occurrence of each *i*, *j* pair. For sidechain-sidechain van-der Waals scoring matrix, the scores are high (yellow blocks) if large-aliphatic residues (i.e. Val, Leu, Ile, and Met) interact to large-aliphatic residues or aromatic residues (i.e. Phe, Tyr, and Trp) interact to aromatic residue. In contrast, the scores are low (orange blocks) when nonpolar residues interact to polar residues. The top two highest scores are 3.0 (Met. interacting to Met) and 2.9 (Trp interacting to Trp).

The value of *E_sim_* was calculated from the BLOSUM62 matrix [31] based on two alignments between two chains (A and B) of the template and their homologous proteins (A' and B'), respectively. The *E_sim_* is defined as(2)

where *CP* is the number of contact residue pairs in the template; *i* and *j* are the contact residue in chains A and B, respectively. *K_ii'_* is the score of aligning residue *i* (in chain A) to *i'* (in protein A') and *K_ji'_* is the score of aligning residue *j* (in chain B) to *j'* (in protein B') according to BLOSUM62 matrix. *K_ii_* and *K_jj_* are the diagonal scores of BLOSUM62 matrix for residues *i* and *j*, respectively. The couple-conserved residue score (*E_cons_*) was determined from two profiles of the template and is given by(3)

where *CP* is the number of contact residue pairs; *M_ip_* is the score in the PSSM for residue type *i* at position *p* in Protein *A; Mp* is the score in the PSSM for residue type *j* at position *p'* in Protein B, and *K_ii_* and *K_jj_* are the diagonal scores of BLOSUM62 matrix for residue types *i* and *j,* respectively.

To evaluate statistical significance (Z-value) of the interacting score of a protein-protein interaction candidate, we randomly generated 10,000 interfaces by mutating 60% contact residues for each heterodimer in 3D-dimer template library. The selected residue was substituted with another amino acid residue according to the probability derived from these 621 complexes [32]. The mean and standard deviation for each 3D-dimer were determined from these 10,000 random interfaces which are assuming to form a normal distribution. Based on the mean and standard deviation, the Z-value of a protein-protein candidate predicted by this template can be calculated.

### Difference between 3D-interologs and previous works

Some enhancements and modifications were applied to the DAPID database [23] and the 3D-partner server [13], thereby improving the reliability and applicability of the 3D- interologs method. There are five main differences between the 3D-interologs and our previous works (Table 1). First, 3D-interologs and 3D-partner integrates knowledge-based scoring matrices and couple-conserved residue scores for measuring binding affinity and interface evolution of homologous PPIs to replace the homologous score in DAPID. Second, 3D-interologs considered the homolog families A' and B' of chains A and B of a template as significant sequence similarity BLASTP E-value ≤10^-10^. The threshold is *E*-value ≤10^-2^ applied in the 3D-partner server and DAPID utilized the *E*-value as the scoring function. Third, 3D-interologs utilized a new method for scoring the template interface similar score. Furthermore, 3D-interologs added a top ranked strategy for a specific species whose genome is deciphered completely. Finally, 3D-interologs and DAPID are databases, conversely, 3D- partner is a web-based service for identifying interacting partners of a query protein sequence.

**Table 1:** The essential differences of DAPID, 3D-partner and 3D-interologs

Feature/Methods	DAPID [23]	3D-partner [13]	3D-interologs
Homolog families of 3D- dimer template	NO	YES (BLASTP *E-value* <10^-2^)	YES (BLASTP *E-value* ≤10^-10^)
**Scoring function:**			
Template interface score	NO	NO	YES (*E_sηm_*)
Top ranked strategy	NO	NO	YES (Top ranked *N* for each species)
Interface binding affinity	NO	YES (four matrices)	YES (four matrices)
Residue conservation score	NO	YES (PSSM)	YES (PSSM)
Score of aligned contact
residues	YES (BLOSUM62)	YES (BLOSUM62)	YES (BLOSUM62)
Service type	A database of domain-annotated protein interactions	A web tool predicts interacting partners and binding models of a query protein sequence	An evolution database of physical protein-protein interactions across multiple genomes

### Inputs and outputs

The 3D-interologs database server is easy-to-use. Users input the UniProt AC or the FASTA format of the query protein (Fig. 2A). The server generally returns a list of interacting partners with functional annotations (e.g. the gene name, the protein description and GO annotations) (Fig. 2D) and provides the visualization of the binding model and contact residues between the query protein and its partner by aligning them to respective template sequences and structures. Additionally, the 3D-interologs system indicates the interacting evolution analysis by using multiple sequence alignments of the interologs across multiple species (Fig. 2C). The significant contact residues in the interface are indicated. If Java is installed in the user's browser, then the output shows the structures, and users can dynamically view the binding model, interacting domains and important residues in the browser.

## Results

### Database

The 3D-interologs database currently contains 15,124 species and 283,980 protein-protein interactions, including 173,294 interactions (61%) derived from our method based on 3D- domain interolog mapping and 110,686 interactions (39%) summarized from the IntAct database [3]. For the hit interacting partner derived from 3D-domain interolog mapping, this database provides functional annotations (e.g. UniProt AC, organism, descriptions, and Gene Ontology (GO) annotations [28]), and the visualization of the binding models and interaction evolutions between the query protein and its partners. On the other hand, the 3D-interologs database presents only the functional annotations of the hit protein-protein interaction if this interaction was summarized from the IntAct database.

Among 15,124 species in the 3D-interologs database, Table 2 shows 19 species commonly used in molecular research projects, such as *Homo sapiens, Mus musculus, Rattus norvegicus, Drosophila melanogaster, Caenorhabditis elegans, Saccharomyces cerevisiae,* and *Escherichia coli.* To analyze couple-conserved residues and interface evolutions for providing evolutionary clues, the 15,124 species were divided into 10 taxonomic groups [33], namely mammalia, vertebrata, metazoa, invertebrata, fungi, plant, bacteria, archaea, viruses, and others.

**Table 2:** Statistics of 3D-interologs database on 19 species commonly used in research projects

Species	3D-domain interologs	IntAct
*Mus musculus*	8,876	2,634
*Homo sapiens*	8,639	18,716
*Danio rerio*	4,564	0
*Xenopus laevis*	4,057	58
*Rattus norvegicus*	3,685	958
*Bos taurus*	3,549	174
*Drosophila melanogaster*	2,644	25,036
*Arabidopsis thaliana*	2,418	2,111
*Caenorhabditis elegans*	1,433	4,684
*Saccharomyces cerevisiae*	443	36,821
*Escherichia coli*	426	14,007
*Schizosaccharomyces pombe*	371	341
*Dictyostelium discoideum*	284	84
*Zea mays*	219	0
*Oryza sativa*	193	69
*Takifugu rubripes*	191	0
*Chlamydomonas reinhardtii*	122	14
*Plasmodium falciparum*	68	2,707
*Pneumocystis carinii*	23	0
other species	131,089	2,272

Total	173,294	110,686

### Example analysis

**Figure 4:**
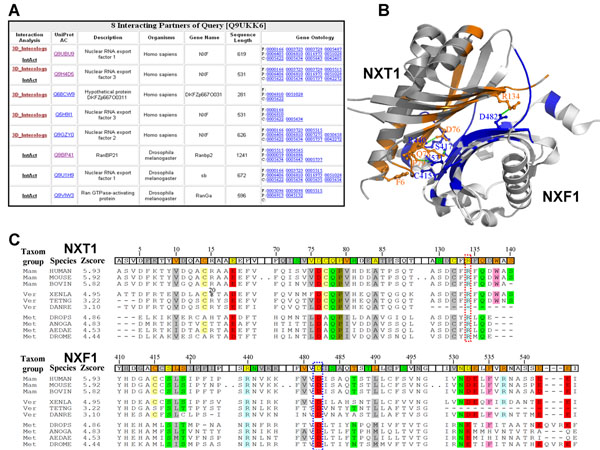
The 3D-interologs database search results of using human NXT1 (UniProt accession number Q9UKK6) as query. (A) Eight interacting partners of NXT1 are found in the 3D-Interologs. For each interacting partner, this server provides UniProt accession number, protein description, organism and Gene Ontology annotation. (B) Detailed interactions between the query and its interacting partner (UniProt accession number Q9UBU9) are indicated via the structure template which consists of NXT1 (PDB entry 1jkg-A) and NXF1 (PDB entry 1jkg-B). The contact residues of NXT1 (query side) and NXF1 (partner side) are colored by red and blue, respectively. The contact residues forming hydrogen bonds (green and dash) are given the atom details. (C) The interacting evolution analysis by using multiple sequence alignments of hit interacting partners of the query across multiple species. The 3D- interologs yields 10 interologs of the query template structure. The contacted residues are marked in template structure based on their interacting characteristics, including hydrogen-bond residues (green); conserved residues (orange); both (yellow), and others (gray). The couple-conserved contact positions are colored in the multiple alignments according to the physical-chemical property of amino acid residues. Twenty amino acid types are classified into 7 groups, namely polar positive (His,Arg, and Lys, blue); polar negative (Asp and Glu, red); polar neutral (Ser, Thr, Asn and Gln, green); cystein (yellow); non-polar aliphatic (Ala, Val, Leu, Ile and Met, gray); non-polar aromatic (Phe, Tyr and Trp, pink); and others: (Gly and Pro, brown).

Figure 4 show the search results using the human protein NXT1 (UniProt AC Q9UKK6) [34] as the query sequence. The NXT1, which is a nucleocytoplasmic transport factor and shuttles between the nucleus and cytoplasm, accumulates at the nuclear pore complexes. For this query, 3D-interologs database yielded 8 hit interacting partners (Fig. 4A), comprising 5 partners derived from 3D-interologs database and 5 partners from the IntACT database. Thus, two partners were present in both databases. Among these 8 hits, 3 partners (i.e. Uniprot AC Q68CW9, Q5H9I1 and Q9GZY0) were not recorded in IntAct database, but they very likely interact with NXT1. The Q68CW9, which is part of the protein NXF1 (UniProt AC Q9UBU9), consists of the UBA-like domain and the NTF-like domain, which is responsible for association with the protein NXT1 [35]. The sequence of the protein Q5H9I1 is the same as that of the protein Q9H4D5 (i.e. nuclear RNA export factor 3), which binds to NXT1 [36]. The protein Q9GZY0 (nuclear RNA export factor 2) binds protein NXT1 to export mRNA cargoes from nucleus into cytosol [37].

The protein NXT1 interacts with the protein NXF1 to form a compact heterodimers (PDB code 1jkg [38])and an interacting β surface, which is lined with hydrophobic and hydrophilic residues (Fig. 4B). Twenty hydrogen bonds or electrostatic interactions are formed in this compact interface. The salt bridge formed by NXT1 Arg134 and NXF1 Asp482 is especially important in the interface [29]. The interacting evolution analysis built by 10 interologs reveals that two residues (Arg134 and Asp482) are conserved in all species (Fig. 4C). Additionally, some interacting residues forming the hydrogen bonds are also couple- conserved, for example NXT1 Asp76 and NXF1 Arg440; NXT1 Gln78 and NXF1 Ser417; NXT1 Pro79 and NXF1 Asn531 [29]. The evolution of interaction is valuable to reflect both couple-conserved and critical residues in the binding site.

Conversely, some positions, which are not conserved in all species but conserved in an individual taxonomic group, are important for observing the co-evolution across multiple species. The interacting residue pair (NXT1 Phe6 and NXF1 Cys415) in mammalia and vertebrata is different from that in metazoan (NXF1 Cys415→Met and NXT1 Phe→Leu variant). The van-der Waals potential (1.3 in the sidechain-sidechain van-der Waals scoring matrix, Fig. 3A) between Leu and Met is much larger than the potential (−0.1) between Cys and Phe. This co-evolution favors the formation of the hydrophobic interaction in metazoan.

### Binding affinity prediction

**Figure 5:**
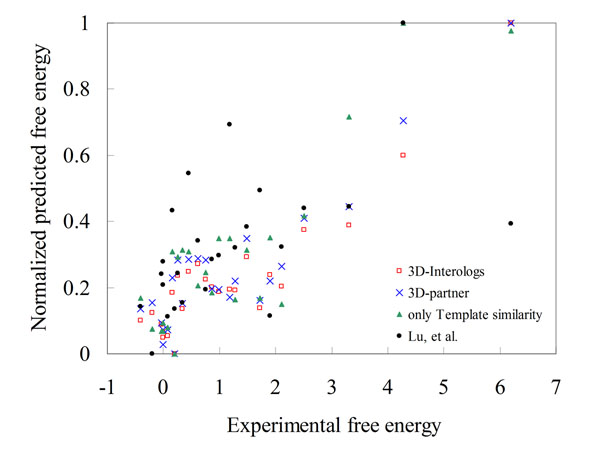
**Evaluation of the 3D-interologs in binding affinities**. The Pearson correlations between experimental free energies (ddG) and the predicted values of the 3D-interologs using four scoring functions, including 3D-interologs (red), 3D-partner (blue), *E_sim_* (only template similarity, green) and one matrix (black) proposed by Lu, *et al.,* on 275 mutated residues selected from Alanine Scanning Energetics database.

The enhanced scoring functions were first evaluated on 275 mutated residues selected from the ASEdb database [29] to reveal the Pearson correlations between ddG values and predicted energies. The 3D-interologs method applied four scoring functions (Fig. 5), including 3D- interologs (red), 3D-partner (blue), *E_sim_* (only template similarity, green) and one matrix (black) proposed by Lu, *et al.*[20]. Among these four scoring functions, the 3D-interologs is the best (0.92) and one matrix is the worst (0.55, i.e. Lu, *et al.*)*.* The correlations are 0.91 and 0.88 for 3D-partner and 0.88 (only template similarity), respectively.

The binding free energy is often not evenly distributed across interfaces but involves a small subset of “hot spots” contributed extraordinarily high energy [39]. For instance, the human blood-coagulation complex (PDB code 1dan) has 52 residues whose energy contribution was probed by alanine scanning mutagenesis [40,41]. Among these 52 residues, residues Lys-20 and Asp-58, which are highly conserved in many species, provide the binding free energy upper 2 kcal/mol; on the other hand, the average energy contribution of the other 50 residues is 0.37 kcal/mol. This result implies that the couple-conserved residue score (*Econs*) is beneficial to model the binding energy of residues positioned in the interfaces. Although the hotspots of protein-protein binding are often for maintaining their function, the antibodies keep the diversity to recognize a wide variation of antigens. The correlation is 0.143 when the *E_cons_* was used to model the binding energy of antigen-antibody complexes. Fortunately, integrating *E_cons_, E_sim_* and *E_SF_* is able to improve the correlation to 0.606 for antigen-antibody complexes.

### Interactions prediction in *S. cerevisiae*

**Figure 6:**
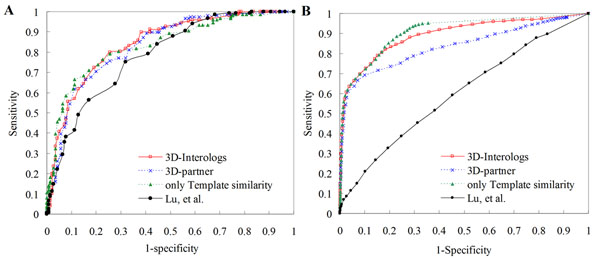
**The ROC curves of the 3D-interologs for protein-protein interactions**. The 3D-interologs search results on (A) *S. cerevisiae* and (B) 549 species (Intger8) using the data set NR-563 (563 dimer-complex structures) by applying four scoring functions, including 3D-interologs (red), 3D-partner (blue), only template similarity (*E_sim_,* green) and one matrix (black) proposed by Lu, *et al.*

Additionally, a non-redundant set (NR-563), comprising 563 dimer complexes from the 3D-dimer library, was adopted to evaluate the performance of this enhanced scoring function for interacting partner predictions in *S. cerevisiae.* This set comprised 5,882 protein-protein interactions, which were recorded as the core subset in the DIP database as the positive cases, and 2,708,746 non-interacting protein pairs, defined by Jansen *et al.*[7] as the negative cases. Figure 6A shows the ROC curves of our method and other three scoring functions for predicting PPIs in *S. cerevisiae.* Among these four scoring functions, the 3D-interologs and the template similar score (*E_sim_*) were the best and achieved the similar accuracy. Conversely, one matrix (i.e. Lu, *et al.*[20]) was the worst. The average precisions, which was calculated as , where  denotes the number of compounds in a hit list including *i* correct hits, were 0.84 (3D-interologs), 0.82 (3D-partner), and 0.67 for one matrix (proposed by Lu *et al*.). These results demonstrated that the proposed new scoring function can achieve good agreement for the binding affinity in PPIs and provide statistical significance (*Z*-value) for predicting PPIs.

### Interactions prediction on multiple species

**Figure 7:**
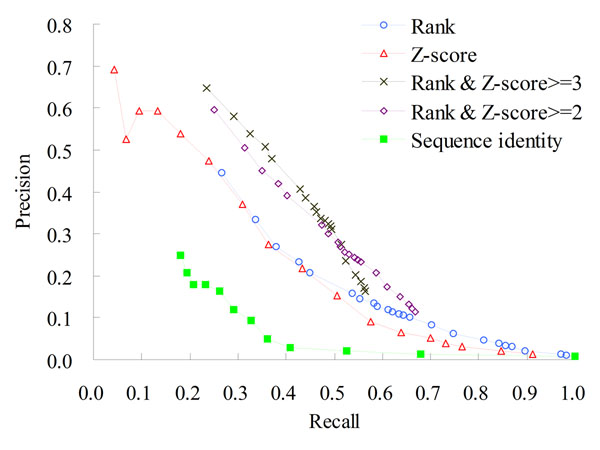
**Precisions and recalls of 3D-interologs the on Integr8**. The 3D-interologs searches Integr8 database (2,102,196 proteins in 549 species) using the data set NR-563 (563 dimer-complex structures). The 3D-interologs server uses five scoring schemes, including rank in a species (blue), Z-score (red), rank and Z-score >=3 (black), rank and Z-score >=2 (purple), and sequence identity (green).

To evaluate the performance of the 3D-domain interolog mapping on multiple species, 563 non-redundant dimer complexes (NR-563) were used as queries to search on the Integr8 database (Release 65) which comprises 2,102,196 proteins in 549 species (Fig. 6B and Fig. 7). The Integr8 is an integrated database for organisms with completely deciphered genomes, which are mainly obtained from the non-redundant sets of UniProt entries. Experimentally determined protein-protein interactions dataset were collected from IntAct [3] as the gold standard positive set (110,686 interactions). The gold standard negative set was generated according to the assumption that two proteins acting in the same biological process are more likely to interact than two proteins involved in different processes [42]. This study applied the relative specificity similarity (RSS), proposed by Wu *et al.*[43], to measure the biological process similarity and the location similarity of two proteins based on the GO terms of the biological process (BP) and the cellular component (CC), which describes locations at levels of subcellular structures and macromolecular complexes, respectively. Among 110,686 interactions recorded in the IntAct database, 51,049 interactions can be used to calculate the BP and the CC RSS scores. The BP and CC RSS scores of 15.85% and 2.65% interactions, respectively, are less than 0.4. Here, we considered an interacting protein pair as a negative PPI if its CC RSS score is less than 0.4.

The structures in the NR-563 as queries to search the Integr8 database yielded 1,063 protein-protein interactions recorded in the IntAct database and 131,831 protein pairs, whose CC RSS scores were less than 0.4 as the negative cases. Based on ROC curves (Fig. 6B) for predicting PPIs in 549 species, 3D-interologs and the template similar score (*E_sim_*) outperform the 3D-partner server and one-matrix (i.e. Lu, *et al*.) method. In addition, the precision and recall were adopted to access the predicted quality of the 3D-interologs using these four scoring schemes (Fig. 7). The precision was defined as *A_h_*/(*A_h_*+*F_h_*), where *A_h_* and *F_h_* denote the numbers of hit positive cases and hit negative cases, respectively. The recall was defined as *A_h_*/*A*, where *A* is the total number of positives (here *A*=1,063). Furthermore, the accuracy of our scoring function (red) is significantly better than that of the sequence identity (green).

The 3D-domain interolog mapping may yield many PPI candidates (e.g. > 200) for one species from a structure template because a eukaryote genome frequently contains multiple paralogous genes. Here, we proposed a top-rank strategy to limit the number of PPIs inferred from a structural template in the same species. For example, we discarded the PPI candidates whose ranks ≥ 25 for a species if the rank threshold is set to 25. Figure 7 shows that the performance of the top-rank scores (blue, with different rank thresholds) is similar to that of using Z-score scoring method (red). When we combined the top-rank strategy and the Z-score scoring methods, the precisions (purple and black) are significantly improved. The precision was 0.52 and the recall was 0.34 when Z-score > 3.0 and the rank ≤25 in one species.

Adopting the top-rank strategy in one species as the scoring function is useful for distinguishing between positives and negatives when the 3D-domain interolog mapping yielded many protein-protein interactions for one species from a structure template. However, the rank cannot reflect the binding affinity of a PPI candidate, conversely, the Z-score cannot be adopted to identify the orthologs and in-paralogs arising from a duplication event following the speciation [27]. These results reveal that Z-scores and ranks scoring methods are complementary.

Table 3 shows an example for illustrating processes and robustness of combining the top- ranked strategy and Z-score methods. Using human calcineurin heterodimer (PDB code 1aui) structure as query, the 3D-domain interolog mapping yielded 1096 PPI candidates in 38 species if the Z score is set to 2. These 1096 candidates possess the interacting domains (i.e. Metallophos and efand domains) of the query template. Among these PPI candidates, 10 PPIs were recorded in IntACT and 9 candidates were considered as negative PPIs because their CC RSS scores are less than 0.4. The ranks of these 9 negative PPIs are more than 15; conversely, these 10 positive PPIS are top 10 in each species. These observations showed that the top- ranked strategy is useful to dramatically reduce the false positive rate when the 3D-domain interolog mapping for predicting PPIs across multiple complete genomes.

**Table 3:** 3D-interologs search results using human calcineurin heterodimer as the query

Interactor1	Interactor2	Species	Z score	Rank	P / N^a^	**RSS of BP**^b^	RSS of CC^c^	Interacting domain1	Interacting domain2
P48456	P48451	Fruit fly	8.98	1	P	0.89	0.85	Metallophos	efand
P23287	P25296	Yeast	8.25	1	P	0.88	1.00	Metallophos	efand
P14747	P25296	Yeast	7.95	2	P	0.88	1.00	Metallophos	efand
Q12705	Q9UU93	Yeast	7.94	1	P	**- ^d^**	0.78	Metallophos	efand
P48456	P47948	Fruit fly	4.42	**16**	**N**	0.41	0.30	Metallophos	efand
P48456	P47949	Fruit fly	4.38	**17**	**N**	0.41	0.30	Metallophos	efand
P48456	P49258	Fruit fly	3.99	**23**	P	0.41	0.56	Metallophos	efand
P48456	Q9VQH2	Fruit fly	3.94	**25**	**N**	0.49	0.33	Metallophos	efand
Q8IAM8	P62203	Plasmdium falciparum	3.79	2	P	-	-	Metallophos	efand
P48456	P48593	Fruit fly	3.72	**31**	P	0.35	0.56	Metallophos	efand
P48456	A1ZAE1	Fruit fly	3.59	**34**	**N**	0.00	0.30	Metallophos	efand
Q27889	P48593	Fruit fly	3.42	**40**	P	-	-	Metallophos	efand
P23287	P06787	Yeast	3.36	5	P	0.61	0.88	Metallophos	efand
P48456	Q9VMT2	Fruit fly	3.03	**50**	**N**	0.41	0.30	Metallophos	efand
P48456	Q7K860	Fruit fly	2.99	**53**	**N**	0.41	0.30	Metallophos	efand
P14747	P06787	Yeast	2.86	6	P	0.61	0.88	Metallophos	efand
P48454	Q9NP86	Human	2.33	**90**	**N**	-	0.00	Metallophos	efand
Q08209	Q9NP86	Human	2.31	**91**	**N**	0.41	0.00	Metallophos	efand
P16298	Q9NP86	Human	2.31	**91**	**N**	-	0.00	Metallophos	efand

3D-interologs infers 10 positive and 9 negative protein-protein interactions by human calcineurin heterodimer (PDB code 1aui), including calmodulin-dependent calcineurin A subunit alpha isoform (chain A with interacting domain Metallophos) and calcineurin subunit B type 1 (chain B with interacting domain efhand), searching on Integr8 database. ^a^ PPI is a positive (P, recorded in IntACT database) or negative case (N, RSS of cellular component is less than 0.4). ^b,c^ The relative specificity similarity (RSS) scores, proposed by Wu *et al.*[43], of Gene Ontology biological process (BP) and cellular component (CC), respectively. ^d^ The protein pair is without Gene Ontology annotations in BP or CC.

## Conclusions

This work demonstrates that the 3D-interologs database is robust and feasible for the interacting evolution of PPIs and DDIs across multiple species. This database can provide couple-conserved residues, interacting models and interface evolution through 3D-domain interolog mapping and template-based scoring functions. The scoring function achieves good agreement for the binding affinity in protein-protein interactions. We believe that the 3D- domain interolog mapping should be useful in protein-protein interacting evolution and is able to infer reliable physical protein-protein interactions across multiple genomes.

## Competing interests

The authors declare that they have no competing interests.

## Authors' contributions

Conceived and designed the experiments: YSL and JMY. Performed the experiments and analyzed the data: YSL, YCC and JMY. Contributed reagents/materials/analysis tools: YSL, YCC and JMY. Wrote the paper: YCC and JMY
